# A New Robust Adaptive Filter Aided by Machine Learning Method for SINS/DVL Integrated Navigation System

**DOI:** 10.3390/s22103792

**Published:** 2022-05-17

**Authors:** Jiupeng Zhu, An Li, Fangjun Qin, Lubin Chang

**Affiliations:** Department of Navigation Engineering, Naval University of Engineering, Wuhan 430033, China; zjp980523@126.com (J.Z.); lian196101@126.com (A.L.); changlubin@163.com (L.C.)

**Keywords:** SINS/DVL integrated system, support vector regression, Variational Bayesian adaptive Kalman filter, robust and adaptive

## Abstract

As an important means of underwater navigation and positioning, the accuracy of SINS/DVL integrated navigation system greatly affects the efficiency of underwater work. Considering the complexity and change of the underwater environment, it is necessary to enhance the robustness and adaptability of the SINS/DVL integrated navigation system. Therefore, this paper proposes a new adaptive filter based on support vector regression. The method abandons the elimination of outliers generated by Doppler Velocity Logger (DVL) in the measurement process from the inside of the filter in the form of probability density function modeling. Instead, outliers are eliminated from the perspective of external sensors, which effectively improves the robustness of the filter. At the same time, a new Variational Bayesian (VB) strategy is adopted to reduce the influence of inaccurate process noise and measurement noise, and improve the adaptiveness of the filter. Their advantages complement each other, effectively improve the stability of filter. Simulation and ship-borne tests are carried out. The test results show that the method proposed in this paper has higher navigation accuracy.

## 1. Introduction

As an important tool for human exploration of the ocean, autonomous underwater vehicles (AUV) have played an irreplaceable role in tasks such as resource exploration and hydrological survey. However, with the increasing complexity of various tasks, new requirements have been placed on the capabilities of autonomous underwater vehicles. High-precision navigation and positioning capability is one of them. Due to the lack of high-precision GNSS signals in the underwater environment, scientists have turned to other means of navigation [1,2,3,4]. At present, the commonly used navigation methods in the underwater environment are mainly divided into: inertial navigation, geophysical navigation, acoustic navigation, cooperative navigation and integrated navigation [5,6].

Strapdown Inertial Navigation System (SINS) is a device that can provide navigation parameters autonomously. It has the advantages of small size, low cost, and easy maintenance. However, when it is used for a long time, errors will be divergent. Seriously affect the navigation accuracy [7,8,9]. Geophysical navigation mainly relies on geographic information such as gravity field and magnetic field for navigation, which has the advantages of high accuracy and ignoring regional restrictions. However, high-precision measurement equipment is required, so it is not widely used [10,11,12]. Acoustic navigation uses the location information provided by hydrophones for navigation, but it is still restricted by itself, resulting in problems such as limited navigation range [13,14]. As a newly emerging navigation method, collaborative navigation is still in the stage of theoretical verification and research [15]. Compared with the above navigation methods, integrated navigation has become the mainstream navigation method with the advantages of low cost and high precision. At present, the commonly used underwater integrated navigation method is mainly the combination of Strapdown Inertial Navigation System and Doppler Velocity Log (DVL) [16,17]. Since DVL is more flexible and autonomous, it can provide relatively accurate three-dimensional velocity. The SINS/DVL integrated navigation method takes the SINS as the main body, and uses the three-dimensional velocity provided by the DVL to correct the errors of the SINS to achieve the purpose of improving the navigation accuracy [18,19].

In the use of SINS/DVL integrated navigation system, data fusion method is the focus of current research. As the basic data fusion method, Kalman filter is very important in integrated navigation system. Since the system model of the SINS/DVL integrated navigation system usually uses linear model, it just matches the Kalman filter. In this case, both the process noise and the measurement noise are required to be Gaussian distributed [20,21]. In practice, many system models are nonlinear, so the EKF and UKF methods are proposed [22,23,24]. The above methods all require the system model to be accurate, but in practical applications, due to extreme conditions such as a large angle motion, the statistical laws of process noise and measurement noise no longer obey the Gaussian distribution. It even leads to outliers in the DVL measurement process, which will seriously affect the accuracy of the integrated navigation system. To solve the problem of time-varying noise and outliers, scholars have proposed some new Kalman filters. Sage–Husa AKF (SHAKF) is a covariance matching method that recursively estimates noise statistics, but cannot guarantee convergence to an accurate noise matrix, causing the filter to diverge [25,26]. In addition, adaptive filters (AKF) can also estimate measurement noise, such as multi-model AKF (MMAKF). Its estimation is completed by using multiple filters, which makes the algorithm have poor real-time performance and a large amount of calculation [27]. Similarly, as an effective method for the uncertainty of system model and noise statistical characteristics, robust filtering is also widely used [28,29], but the selection of constraints limits its use. Some outliers may not be fully filtered. In addition, the adaptive filter based on Variational Bayesian theory can also estimate the measurement noise, the method estimates the measurement noise by choosing appropriate prior distributions such as the inverse Gamma distribution and the inverse Wishart distribution. Although this method can achieve adaptation to measurement noise, the premise is that the accurate statistical distribution of process noise is known [30,31]. The above method can effectively improve the adaptability of the filter, but when outliers appear in the measured values, the performance of filter will be greatly degraded, so it is also important to improve the robustness of the filter. Ref. [32] proposes a novel robust filter based on Student’s T distribution (RSTKF) to address thick-tailed process noise and measurement noise. Ref. [33] proposes a robust Kalman filter (ODKF) with outlier detection, which utilizes a binary indicator variable to mark outliers and eliminate them. Ref. [34] proposes a robust filtering algorithm based on Mahalanobis distance (HRAKF). The above three methods can eliminate outliers, but the adaptive ability is reduced. Ref. [35] proposes a new adaptive robust method, but the amount of calculation is increased, and the probability of singular values generated by matrix decomposition will be increased.

**Figure 1:**
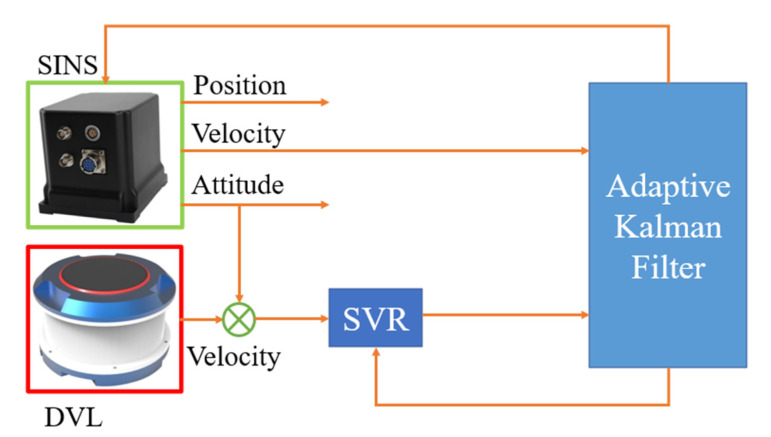
Schematic diagram of SINS/DVL integrated navigation system.

From the above analysis, it is not difficult to find that it is very important to develop a method that can solve time-varying noise and the outliers in the process of measurement at the same time. Ref. [36] proposes a new adaptive filter based on Variational Bayesian theory, it can estimate the process noise and measurement noise simultaneously according to the Variational Bayesian method without relying on the precise process noise, which effectively improves the adaptive ability. At the same time, in order to enhance the robustness of the method, but not excessively affect the calculation speed and complexity of matrix, it is necessary to seek a new robustness method. With the continuous development of machine learning, this kind of problem can be solved. As a machine learning method that can achieve prediction, support vector regression (SVR) can be well combined with adaptive methods. By detecting and eliminating outliers of external sensors, the robustness of the filter is effectively improved without affecting the performance of the filter, and the anti-interference ability of the integrated navigation system is effectively enhanced. Therefore, the proposed adaptive filter based on VB theory assisted by support vector regression can enhance the adaptability and robustness of filter at the same time. As shown in Figure 1.

The organization of this paper is as follows. The second part briefly introduces the basic model of the SINS/DVL integrated navigation system. The third part introduces the Variational Bayesian adaptive filter aided by support vector regression proposed in this paper, and gives the specific flow of the algorithm. In the fourth section, simulation experiments are carried out to evaluate the performance of the proposed method. In the fifth part, the shipboard test is carried out to further illustrate the advantages and feasibility of the algorithm. Finally, a few meaningful conclusions are outlined in the summary.

## 2. Principle of SINS/DVL Integrated Navigation System

**Figure 2:**
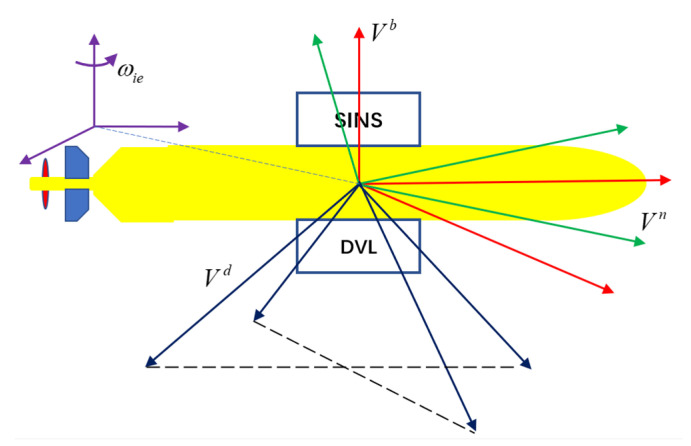
Schematic diagram of each reference coordinate frame. The green line represents the navigation coordinate frame, the red line represents the carrier coordinate frame, and the blue line represents the DVL device coordinate frame.

In this paper, the following frames are specifically defined: the earth-centered inertial coordinate frame is represented by *i*, the body coordinate frame of the SINS is represented by *b*, the coordinate frame for the SINS to solve the navigation parameters is represented by *n*, *d* denotes the DVL instrument coordinate frame. In the process of integrated navigation, the SINS measures the specific force and angular velocity under the *b* frame, and converts them into the *n* frame through attitude conversion and integral operation. However, what DVL gets is the velocity of the carrier in the *d* frame, so two attitude transformations are required to convert it into the velocity of the *n* frame [37]. For specific reference frames, please refer to Figure 2 below:

The linear error equations of the attitude, velocity, and position of the SINS are directly given here, where the attitude errors are expressed as ***φ***, the velocity errors are expressed as $\delta v^{n}$, and the position errors are expressed as δp.

(1) $$\begin{array}{c} \dot{\varphi }\;=\;M_{aa}\varphi +M_{av}\delta v^{n}+M_{ap}\delta p-C_{b}^{n}\varepsilon^{b}\\ \delta {\dot{v}}^{n}\;=\;M_{va}\varphi +M_{vv}\delta v^{n}+M_{vp}\delta p+C_{b}^{n}\nabla^{b}+\delta g^{n}\\ \delta \dot{p}\;=\;M_{pv}\delta v^{n}+M_{pp}\delta p \end{array}$$

In the Equation (1), $\varepsilon^{b}$ and $\nabla^{b}$ represent the measurement errors of the gyroscopes and accelerometers under the carrier system, and the matrix of each errors conversion involved is shown in the Equation (2).

(2) $$\begin{array}{cc} M_{aa}= & -\left(\omega_{in}^{n}\times \right)\;M_{av}=\left[\begin{array}{ccc} 0 & -1/R_{Mh} & 0\\ 1/R_{Nh} & 0 & 0\\ \tan L/R_{Nh} & 0 & 0 \end{array}\right]\\ M_{ap}= & \left[\begin{array}{ccc} 0 & 0 & v_{N}^{n}/R_{Mh}^{2}\\ -\omega_{ie}\sin L & 0 & -v_{E}^{n}/R_{Nh}^{2}\\ \omega_{ie}\cos L+v_{E}^{n}\sec^{2}L/R_{N} & 0 & -v_{E}^{n}\tan L/R_{Nh}^{2} \end{array}\right] \end{array}$$

In the Equation (4), $R_{Mh}=R_{M}+h$, $R_{Nh}=R_{N}+h$. $R_{M}$, $R_{N}$, h represent the meridian and prime vertical radius of the earth and height. $v_{N}^{n}$, $v_{E}^{n}$ represent east and north velocity.$\omega_{ie}^{n}$ represents the angular velocity of earth rotation. $\omega_{in}^{n}$ in $M_{aa}$ can be described as $\omega_{in}^{n}=\omega_{ie}^{n}+\omega_{en}^{n}$. The expression of $\omega_{en}^{n}$ is described as Equation (3).

(3) $$\omega_{en}^{n}={\left[\begin{array}{ccc} -\frac{v_{N}^{n}}{R_{M}+h} & \frac{v_{E}^{n}}{R_{N}+h} & \frac{v_{E}^{n}}{R_{N}+h}\tan L \end{array}\right]}^{T}$$

The remaining errors matrix expression are as follows

(4) $$\begin{array}{l} M_{va}=\left(f_{sf}^{n}\times \right)M_{vv}=-\left[\left(v^{n}\times \right)M_{av}+\left(2\omega_{ie}^{n}+\omega_{en}^{n}\right)\times \right]\\ M_{ap}=\left(v^{n}\times \right)\left[\begin{array}{ccc} 0 & 0 & v_{N}^{n}/R_{Mh}^{2}\\ -2\omega_{ie}^{n}\sin L & 0 & -v_{E}^{n}/R_{Nh}^{2}\\ 2\omega_{ie}^{n}\cos L+v_{E}^{n}\sec^{2}L/R_{N} & 0 & -v_{E}^{n}\tan L/R_{Nh}^{2} \end{array}\right] \end{array}$$

where $f_{sf}^{b}$ represents the accelerometer measurement, and the remaining errors matrix are expressed as Equation (5).

(5) $$\begin{array}{c} M_{pv}=\left[\begin{array}{ccc} 0 & 1/R_{Mh} & 0\\ \sec L/R_{Nh} & 0 & 0\\ 0 & 0 & 0 \end{array}\right]\\ M_{pp}=\left[\begin{array}{ccc} 0 & 0 & -v_{N}^{n}/R_{Mh}^{2}\\ v_{E}^{n}\sec L\tan L/R_{Nh} & 0 & -v_{E}^{n}\sec L/R_{Nh}^{2}\\ 0 & 0 & 0 \end{array}\right] \end{array}$$

The state equation in the SINS/DVL integrated navigation system is usually described by the Equation (6).

(6) $$\dot{X}\;=\;\mathrm{FX}+W$$

Here the dimensions of the state variable are 15 dimensions, and its expression is as follows

(7) $$X={\left[\begin{array}{ccccc} \varphi & \delta v^{n} & \delta p & \varepsilon^{b} & \nabla^{b} \end{array}\right]}^{T}$$

In the Equation (7), F is the state transition matrix, its structure is composed of M, W is the state noise, for their specific definitions can refer to [38].

The measurement equations of the integrated navigation system are introduced below. Since DVL first obtains the velocity in its own device system during the measurement process, it is necessary to perform coordinate frame conversion if information fusion is to be performed [39].

(8) $$v_{DVL}^{d}\;=\;{\tilde{C}}_{b}^{n}C_{d}^{b}v_{SINS}^{n}$$

In the Equation (8), $v_{SINS}^{n}$ represents the velocity under the navigation frame, $C_{d}^{b}$ represents the attitude transformation matrix from the *d* frame to *b* frame, and ${\tilde{C}}_{b}^{n}$ represents the attitude transformation matrix from the *b* frame to *n* frame. The expressions of the two attitude change matrices are as Equation (9)

(9) $$\left\{ \begin{array}{l} {\tilde{C}}_{b}^{n}\;=\;\left(I-\varphi \times \right)C_{b}^{n}\\ C_{d}^{b}=I-\eta \times \end{array}\right.$$

Further sorting out, the Equation (10) is established

(10) $$\begin{array}{ll} v_{DVL}^{n} & =\left[I-\varphi \times \right]C_{b}^{n}C_{d}^{b}v_{DVL}^{d}\\ & =C_{b}^{n}C_{d}^{b}v_{DVL}^{d}+\left[\left(C_{b}^{n}C_{d}^{b}v_{DVL}^{d}\right)\times \right]\varphi \\ & =\;{\tilde{v}}_{DVL}^{n}+\delta v_{DVL}^{n} \end{array}$$

In fact, in the process of information fusion, the measured value of the filter is the difference between the velocity value output by SINS and the velocity output by DVL. It can be described as Equation (11).

(11) $$\begin{array}{ll} Z & =v_{SINS}^{n}-v_{DVL}^{n}=\left({\tilde{v}}_{SINS}^{n}+\delta v_{SINS}^{n}\right)-\left({\tilde{v}}_{DVL}^{n}+\delta v_{DVL}^{n}\right)\\ & =\delta v_{SINS}^{n}-\delta v_{DVL}^{n}=\delta v_{SINS}^{n}-\left[\left(C_{b}^{n}C_{d}^{b}v_{DVL}^{d}\right)\times \right]\varphi \\ & =\;\mathrm{HX}\;+\;V \end{array}$$

where H is the measurement matrix and V is the measurement noise matrix. The form of the measurement matrix is as Equation (12).

(12) $$H=\left[\begin{array}{ccc} I_{3\times 3} & \left(C_{b}^{n}C_{d}^{b}v^{d}\right)\times & 0_{3\times 6} \end{array}\right]$$

## 3. Principle of Support Vector Regression Assisted Adaptive Filter

### 3.1. The Principle of Support Vector Regression

The concept of support vector machine (SVM) was first proposed by Cortes and Vapnik based on the theory of statistical learning, and it has been well applied in the field of classification and regression. SVM changes the traditional empirical risk minimization principle, so it has good generalization ability. When the SVM deals with nonlinear problems, it first transforms the nonlinear problem into a linear problem in a high-dimensional space, and then uses the kernel function to avoid the inner product operation in the high-dimensional space, effectively solving the problem of the curse of dimensionality and local minimum. When SVM is used to solve regression problems, it is also called Support Vector Regression (SVR) [40,41]. The principle of support vector regression is introduced below.

Suppose there is a sample set $S=\left\{\left(x_{i},y_{i}\right)\right\}$, where $x_{i}$ and $y_{i}$ are the input and output of the training set. In the high-dimensional feature space, there must be a linear function f that can establish a nonlinear relationship between $x_{i}$ and $y_{i}$. Such a fitting function expression is as Equation (13).

(13) f(x)=ω⋅φ(x)+b

where φ(·) is a nonlinear transformation, which maps the input space to a high-dimensional feature space (Hilbert space), which is convenient for linear approximation in the feature space. ω and *b* are the parameters to be requested. Different from the traditional regression model, SVR is committed to minimizing empirical risk and structural risk. The target model and constraints of the SVR model are as Equation (14).

(14) $$\begin{array}{l} \min\frac{1}{2}{\left\|\omega \right\|}^{2}+C\sum_{i=1}^{n}\left(\xi_{i}+\xi_{i}^{*}\right)\\ s.t.\left\{ \begin{array}{l} y_{i}-f(x_{i})\leq \varepsilon +\xi_{i}\\ f(x_{i})-y_{i}\leq \varepsilon +\xi_{i}^{*}\;i=1,2,\ldots ,n\\ \xi_{i},\xi_{i}^{*}\geq 0 \end{array}\right. \end{array}$$

where n is the number of training samples, $\xi_{i}$ and $\xi_{i}^{*}$ are non-negative slack variable, which is used to measure the deviation from the distance-insensitive boundary.$\frac{1}{2}{\left\|\omega \right\|}^{2}$ is the structural risk, which is used to prevent overtraining. C is the boundary coefficient for balancing structural risk and empirical risk, the selection of its value directly affects the ability of transformation. The basic principle of SVR can be seen in Figure 2.

**Figure 3:**
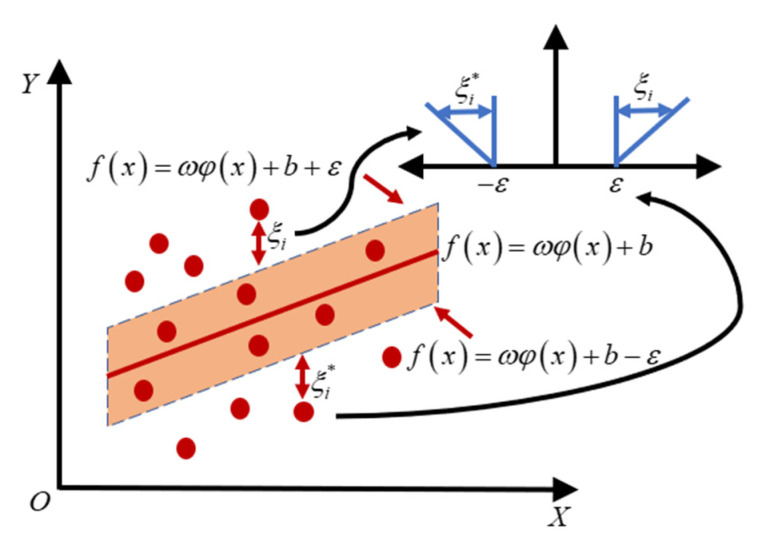
Support vector regression diagram.

In Figure 3, ε is insensitive loss function. If the error between regression prediction result and training sample is less than ε, no error vector will be generated. The sample points outside the prediction model with $\xi_{i}$ will influence the minimizing equation.

By introducing the Lagrangian multiplier method, it is converted to a dual space to solve:

(15) $$\begin{array}{ll} \max\omega (a,a^{*}) & =-\frac{1}{2}\sum_{i,j=1}^{n}\left(a_{i}-a_{i}^{*}\right)(a_{j}-a_{j}^{*})\langle \varphi (x_{i}),\varphi (x_{j})\rangle \\ & -\sum_{i=1}^{n}(a_{i}+a_{i}^{*})\varepsilon +\sum_{i=1}^{n}(a_{i}-a_{i}^{*})y_{i} \end{array}$$

In the Equation (15), $\alpha_{i}$ and $\alpha_{i}^{*}$ are the Lagrange multipliers. According to the Karush-Kuhn-Tucker (KKT) condition, solve and organize the expression of the final regression function at the optimum is as Equation (16).

(16) $$f(x)=\sum_{i=1}^{n}\left(a_{i}-a_{i}^{*}\right)\langle \varphi (x),\varphi (x_{i})\rangle +b$$

Here introduces a kernel function that meets the Mercer condition $k\left(x,x_{i}\right)=\langle \varphi \left(x\right),\varphi (x_{i})\rangle$, then the Equation (16) becomes Equation (17).

(17) $$f(x)=\sum_{i=1}^{n}\left(a_{i}-a_{i}^{*}\right)k(x,x_{i})+b$$

Different kernel functions can construct different support vector machines. Compared with other kernel functions, Radial Basis Function (RBF) has the characteristics of few parameters and less computation. In order to improve the calculation efficiency of real-time compensation, this paper selects RBF as the kernel function of SVR, Equation (18) gives the expression of the function.

(18) $$k(x,x_{i})=\exp({\left\|x-x_{i}\right\|}^{2}/2\sigma^{2})$$

### 3.2. Principle of Adaptive Filter Based on Variational Bayesian Theory

Firstly, the discrete-time linear stochastic system obtained from the state-space model of the integrated navigation system needs to be considered.

(19) $$\begin{array}{l} x_{k}=f\left(x_{k-1}\right)+w_{k-1}\\ y_{k}=h\left(x_{k}\right)+v_{k} \end{array}$$

In the Equation (19), k is discrete time, both process noise $w_{k-1}$ and measurement noise $v_{k}$ obey Gaussian distribution with mean 0 and covariance matrices of $Q_{k-1}$ and $R_{k}$. f(·) and h(·) are both linear. In such a case, the estimation result of KF is optimal. However, in practical applications, the noise is time-varying, and using the noise matrix that does not satisfy the Gaussian distribution will seriously affect the filtering results. Therefore, it is necessary to adopt an adaptive filtering strategy to solve such problems. Traditional adaptive filtering aims to estimate the posterior distribution of state and noise covariance $p\left(x_{k},R_{k}|y_{1:k}\right)$, $y_{1:k}$ represents the collection of measurements from the initial time to time k. Since such a joint probability density function does not have an exact analytical solution, the conventional Variational Bayesian approximation is used to decompose the joint probability density function of the state and measurement noise covariance as follows

(20) $$p\left(x_{k},R_{k}|y_{1:k}\right)\approx \vartheta_{x}\left(x_{k}\right)\vartheta_{R}\left(R_{k}\right)$$

In the Equation (20), $\vartheta_{x}\left(x_{k}\right)$ and $\vartheta_{R}\left(R_{k}\right)$ are the approximate posterior probability density function. But at its root, this assumption is based on a known precise distribution of process noise. In cases where the statistical distribution of process noise is unknown, this can lead to filter performance degradation. Therefore, in order to deal with the problem of inaccurate process noise, the prediction error covariance matrix is added to the original method [39], and the above formula can be reconstructed as

(21) $$p\left(x_{k},P_{k|k-1},R_{k}|y_{1:k}\right)\approx \vartheta_{x}\left(x_{k}\right)\vartheta_{P}\left(P_{k|k-1}\right)\vartheta_{R}\left(R_{k}\right)$$

It can be seen from the In the Equation (21), that the posterior joint probability density function $p\left(x_{k},P_{k|k-1},R_{k}|y_{1:k}\right)$ can be decomposed into approximations of the posterior probability density function after VB approximation. In this way, the problem of finding the joint probability density function is transformed into the approximation of three probability density functions. As for how to obtain these three approximations, it must be clear that the core idea of VB approximation is to minimize the Kullback-Leibler divergence (KLD) between the true distribution and the approximation. The Equation (22) is as follows [42]

(22) $$\begin{array}{l} \left\{\vartheta_{x}\left(x_{k}\right),\vartheta_{P}\left(P_{k|k-1}\right),\vartheta_{R}\left(R_{k}\right)\right\}=\arg\min\mathrm{KLD}\\ \left(\vartheta_{x}\left(x_{k}\right)\vartheta_{P}\left(P_{k|k-1}\right)\vartheta_{R}\left(R_{k}\right)||p\left(x_{k},P_{k|k-1},R_{k}|z_{1:k}\right)\right) \end{array}$$

The Equation (23) is the calculation formula of Kullback-Leibler divergence (KLD)

(23) $$\mathrm{KLD}\left(\vartheta \left(x\right)||p\left(x\right)\right)=\int \vartheta \left(x\right)\log\frac{\vartheta \left(x\right)}{p\left(x\right)}dx$$

After the calculation formula is clear, the optimal solution of the above formula can be obtained [42].

(24) $$\log\left(\vartheta (\lambda )\right)=E_{\Xi^{\left(-\lambda \right)}}\left[\log p\left(\Xi ,y_{1:k}\right)\right]+c\left(\lambda \right)$$

In the Equation (24), E[·] indicates the desired operation, Ξ represents a set of $x_{k}$, $P_{k|k-1}$, $R_{k}$ three elements. λ denotes any one of the elements in Ξ. $\Xi^{-\lambda }$ represents any element in the collection except λ. Since the three approximate posterior probability density functions are coupled to each other, fixed-point iteration is required to solve the above equation. That is, the approximate posterior probability density function ϑ(λ) of any element in the set is updated to $\vartheta^{\left(i+1\right)}\left(\lambda \right)$, which i is the number of fixed-point iteration.

Through the analysis of the above formula, it can be seen that if it is required to obtain ϑ(λ), it is necessary to solve $p\left(\Xi ,y_{1:k}\right)$ first. According to the conditional independence of the hierarchical state space model, the probability density function $p\left(\Xi ,y_{1:k}\right)$ can be decomposed as follows

(25) $$\begin{array}{ll} p\left(\Xi ,y_{1:k}\right) & \;=\;p\left(y_{k}|x_{k},R_{k}\right)p\left(x_{k}|y_{1:k-1},P_{k|k-1}\right)\\ & \;\times \;p\left(P_{k|k-1}|y_{1:k-1}\right)p\left(R_{k}|y_{1:k-1}\right)p\left(y_{1:k-1}\right) \end{array}$$

The first two items of the Equation (25) are not unfamiliar. Since the traditional Kalman filter is processed based on the Gaussian approximate distribution, $x_{k}\sim N\left({\hat{x}}_{k|k-1},P_{k|k-1}\right)$ and $y_{k}\sim N\left(H_{k}x_{k},R_{k}\right)$, Then the one-step prediction probability density function and the probability density function of the measurement error distribution are both Gaussian, so the Equations (26) and (27) holds

(26) $$p\left(y_{k}|x_{k},R_{k}\right)=N\left(y_{k};H_{k}x_{k},R_{k}\right)$$

(27) $$p\left(x_{k}|y_{1:k-1},P_{k|k-1}\right)=N\left(x_{k};{\hat{x}}_{k|k-1},P_{k|k-1}\right)$$

where N(μ,∑) represents the mean μ of the probability density function of the Gaussian distribution, and the covariance matrix is ∑. The true $Q_{k}$ and $R_{k}$ are not available in one-step prediction, Therefore, it is necessary to select appropriate prior distributions for the measurement noise covariance matrix and the prediction error covariance matrix, in order to ensure that the prior distribution and the posterior distribution have the same functional form, such a prior distribution must be conjugate. Inv-Wishart is often used to represent the conjugate prior of the covariance matrix of the Gaussian distribution. The specific form and relevant parameters of Inv-Wishart distribution can refer to reference [43]. Because $P_{k|k-1}$ and $R_{k}$ as shown in the above equations, are both Gaussian covariance matrices, so their prior distributions are selected as Inv-Wishart distributions.

(28) $$p\left(P_{k|k-1}|y_{1:k-1}\right)=IW\left(P_{k|k-1};{\hat{m}}_{k|k-1},{\hat{M}}_{k|k-1}\right)$$

(29) $$p\left(R_{k}|y_{1:k-1}\right)=IW\left(R_{k};{\hat{v}}_{k|k-1},{\hat{V}}_{k|k-1}\right)$$

In the Equation (28) and Equation (29), IW(μ,∑) represents the dof parameter of the Inv-Wishart distribution is μ, and the inverse scale matrix is ∑. Referring to the Equation (30) for calculating the mean value of the Inv-Wishart distribution in [41], there are

(30) $${\hat{M}}_{k|k-1}={\tilde{P}}_{k|k-1}\left({\hat{m}}_{k|k-1}-n-1\right)$$

where n is the dimension of the system state equation, let ${\hat{m}}_{k|k-1}=n+\tau +1$, be the adjustment parameter τ>0. It can be described as Equation (31).

(31) $${\hat{M}}_{k|k-1}=\tau {\tilde{P}}_{k|k-1}$$

According to Bayesian principle, the prior distribution $p\left(R_{k}|y_{1:k}\right)$ can be expressed as the Equation (32)

(32) $$p\left(R_{k}|y_{1:k-1}\right)=\int p\left(R_{k}|R_{k-1}\right)p\left(R_{k}|y_{1:k-1}\right)dR_{k-1}$$

However, it is difficult to determine $p\left(R_{k}|R_{k-1}\right)$, considering that the measurement noise covariance changes slowly in the actual integrated navigation system. Here, the heuristic method proposed in [30] is used to expand the previous approximate posteriors through ρ. The specific process is as follows

(33) $${\hat{v}}_{k|k-1}=\rho \left({\hat{v}}_{k-1}-m-1\right)+m+1$$

(34) $${\hat{V}}_{k|k-1}=\rho {\hat{V}}_{k-1}$$

In the Equations (33) and (34), the value range of the forgetting factor is ρ∈(0,1], and the initial value of the measurement matrix is $\tilde{R}={\hat{V}}_{0}/\left({\hat{v}}_{0}-m-1\right)$.

The Equation (25) can be rewritten as the Equation (35)

(35) $$\begin{array}{ll} p\left(\Xi ,y_{1:k}\right) & =N\left(y_{k}|H_{k}x_{k},R_{k}\right)N\left(x_{k}|{\hat{x}}_{k|k-1},P_{k|k-1}\right)\\ & \times \mathrm{IW}\left(P_{k|k-1}|{\hat{m}}_{k|k-1},M_{k|k-1}\right)\mathrm{IW}\left(R_{k}|{\hat{v}}_{k|k-1},V_{k|k-1}\right)p\left(y_{1:k-1}\right) \end{array}$$

Then the principle of obtaining log(ϑ(λ)) is to obtain $log\left(\vartheta \left(P_{k|k-1}\right)\right)$, $log\left(\vartheta \left(R_{k}\right)\right)$, $log\left(\vartheta \left(x_{k}\right)\right)$ in turn according to the principle that a single variable is gradually introduced. For the specific calculation process can refer to [34], and the specific algorithm flow is given in the next section. At the same time, after fixed-point iterations of times *N*, the variational approximation of the posterior probability density function is given by the Equation (36).

(36) $$\left\{ \begin{array}{l} \vartheta (P_{k|k-1})\approx \vartheta^{\left(N\right)}(P_{k|k-1})\\ \vartheta (R_{k})\approx \vartheta^{\left(N\right)}(R_{k})\\ \vartheta (x_{k})\approx \vartheta^{\left(N\right)}(x_{k}) \end{array}\right.$$

### 3.3. Support Vector Regression Assisted Adaptive Filter Algorithm Specific Process

In the last section, the principle of the adaptive filter based on the VB theory is described, but it cannot be denied that in practical applications, the anti-interference ability of this filter is low, especially in the DVL measurement process. Such outliers can easily lead to filter instability. The idea of the traditional robust filter is to reconstruct the measurement noise matrix based on statistical theory, and to discriminate and eliminate outliers. However, when the number of iterations is too large or it is affected by external non-Gaussian noise, it is easy to cause the problem of inaccurate singular value decomposition or even divergence in the filtering process. Therefore, a method specially designed for outlier elimination of external sensors is adopted to improve the robustness of the integrated navigation system. The specific principles are as follows:

Firstly, the time update can be described as Equation (37):

(37) $$\left\{ \begin{array}{l} {\hat{x}}_{k|k-1}=F_{k-1}{\hat{x}}_{k-1|k-1}\\ {\hat{P}}_{k|k-1}=F_{k-1}P_{k-1|k-1}F_{k-1}^{T}+{\hat{Q}}_{k-1} \end{array}\right.$$

After the time update, the measurement update should have been performed. Here, the DVL data is processed by the SVR method before the measurement update. First, a sliding window of length needs to be established, then at the current moment, the historical measurement data set of external sensors is $\left\{y_{k-L},y_{k-L+1},\cdots ,y_{k-m}\right\}$, when using one of the data for prediction, the Equation (38) mapping relationship can be established, where m is called the model order.

(38) $${\hat{y}}_{k}=\varphi \left(y_{k-1},y_{k-2},\cdots ,y_{k-m}\right)$$

In order to use the SVR algorithm to train the prediction function, the historical data samples of the sensor are reconstructed and transformed into input samples in the form of a matrix. The input and output samples are respectively Y and Z, the specific form is as Equation (39).

(39) $$\left\{ \begin{array}{l} Y=\left[\begin{array}{cccc} y_{k-N} & y_{k-N+1} & \cdots & y_{k-N+m-1}\\ y_{k-N+1} & y_{k-N+2} & \cdots & y_{k-N+m}\\ \vdots & \vdots & \vdots & \vdots \\ y_{k-m} & y_{k-m+1} & \cdots & y_{k-2} \end{array}\right]\\ Z=\left[\begin{array}{c} y_{k-N+m}\\ y_{k-N+m+1}\\ \vdots \\ y_{k-1} \end{array}\right] \end{array}\right.$$

By training the above training samples, the predicted value ${\hat{y}}_{k}$ at the current moment is obtained as shown in the Equation (40)

(40) $${\hat{y}}_{k}=\sum_{i=1}^{N-m}\left(\alpha_{i}-\alpha_{i}^{*}\right)k\left(Z_{i},Z_{k}\right)+b$$

After the predicted value is obtained, it is necessary to carry out the step of discriminating outliers. Here, the innovation $\tilde{y}=H{\hat{x}}_{k|k-1}$ is introduced to discriminate outliers. The specific form is as Equation (41)

(41) $$\Delta y=\left\{ \begin{array}{l} y_{k}-{\tilde{y}}_{k}\geq T\;y_{k}\;\mathrm{is}\;\mathrm{outlier}\;\\ y_{k}-{\tilde{y}}_{k}<T\;y_{k}\;\mathrm{is}\;\mathrm{not}\;\mathrm{outlier}\; \end{array}\right.$$

The selection principle of the discriminant threshold T is usually carried out in accordance with the principle of 3σ, the threshold value can be selected by the difference between the actual value and the predicted value in the sliding window. The average value and standard deviation in the sliding window are defined as Equation (42).

(42) $$\left\{ \begin{array}{l} \delta \bar{y}=\frac{1}{L}\sum_{i=1}^{L}\left(y_{i}-{\hat{y}}_{i}\right)\\ \sigma_{y}=\frac{1}{N-1}\sqrt{\sum_{i=1}^{N}{\left(\delta y_{i}-\delta \bar{y}\right)}^{2}} \end{array}\right.$$

**Figure 4:**
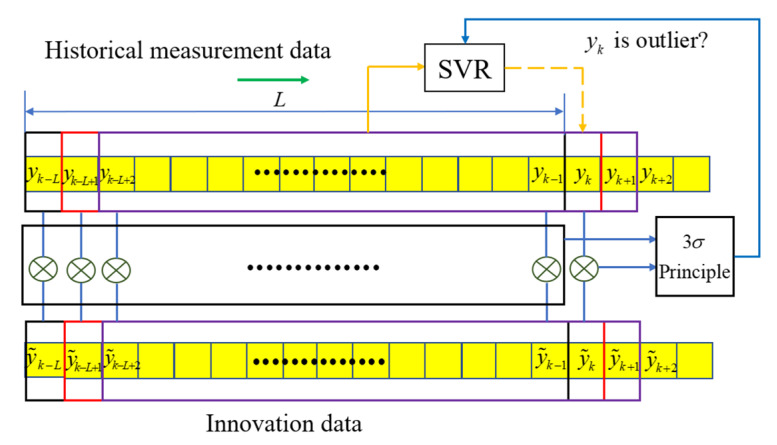
Schematic diagram of Outlier Replacement.

After obtaining the mean and standard deviation of the difference between the actual value and the predicted value in the sliding window, set the threshold $T=3\sigma_{y}$. When the difference between the real value and the innovation Δy is greater than the threshold T, the predicted value is used to replace the real value for measurement update. The specific schematic diagram is shown as Figure 4.

The next step is to perform the measurement update, and the parameters need to be initialized before the update. Equation (43) represents the initialization process.

(43) $$\left\{ \begin{array}{l} {\hat{x}}_{k|k}^{\left(0\right)}={\hat{x}}_{k|k-1},P_{k|k}^{\left(0\right)}={\tilde{P}}_{k|k-1}\\ {\hat{m}}_{k|k-1}=n+\tau +1,{\hat{M}}_{k|k-1}=\tau {\tilde{P}}_{k|k-1}\\ {\hat{v}}_{k|k-1}=\rho \left({\hat{v}}_{k-1|k-1}-m-1\right)+m+1,{\hat{V}}_{k|k-1}=\rho {\hat{V}}_{k-1|k-1} \end{array}\right.$$

Enter the fixed-point iterative update process after initializing the parameter values.

The first is to update the approximate probability density function ${\hat{P}}_{k|k-1}$ of the one-step forecast covariance matrix. Equation (44) gives the calculation process.

(44) $$\left\{ \begin{array}{l} S_{k}^{\left(i\right)}=P_{k|k}^{\left(i\right)}+\left({\hat{x}}_{k|k-1}^{\left(i\right)}-{\hat{x}}_{k|k-1}\right){\left({\hat{x}}_{k|k-1}^{\left(i\right)}-{\hat{x}}_{k|k-1}\right)}^{T}\\ {\hat{m}}_{k|}^{\left(i+1\right)}={\hat{m}}_{k|k-1}+1,{\hat{M}}_{k}^{\left(i+1\right)}=S_{k}^{\left(i\right)}+{\hat{M}}_{k|k-1} \end{array}\right.$$

The approximate probability density function of the imprecise measurement noise covariance matrix $R_{k}$ is then updated by Equation (45).

(45) $$\left\{ \begin{array}{l} T_{k}^{\left(i\right)}=\left(y_{k}-H_{k}{\hat{x}}_{k|k}^{\left(i\right)}\right){\left(y_{k}-H_{k}{\hat{x}}_{k|k}^{\left(i\right)}\right)}^{T}+H_{k}P_{k|k}^{\left(i\right)}H_{k}^{T}\\ {\hat{v}}_{k|}^{\left(i+1\right)}={\hat{v}}_{k|k-1}+1,{\hat{V}}_{k}^{\left(i+1\right)}=T_{k}^{\left(i\right)}+{\hat{V}}_{k|k-1} \end{array}\right.$$

Next, update the approximate probability density function of the state by using the update expression of the approximate probability density function obtained before. The specific calculation process is shown in Equation (46).

(46) $$\left\{ \begin{array}{l} {\hat{P}}_{k|k-1}^{\left(i+1\right)}={\left({\hat{v}}_{k}^{\left(i+1\right)}-m-1\right)}^{-1}{\hat{V}}_{k}^{\left(i+1\right)},{\hat{R}}_{k}^{\left(i+1\right)}={\left({\hat{m}}_{k}^{\left(i+1\right)}-n-1\right)}^{-1}{\hat{M}}_{k}^{\left(i+1\right)}\\ K_{k}^{\left(i+1\right)}={\hat{P}}_{k|k-1}^{\left(i+1\right)}H_{k}^{T}{\left(H_{k}{\hat{P}}_{k|k-1}^{\left(i+1\right)}H_{k}^{T}+{\hat{R}}_{k}^{\left(i+1\right)}\right)}^{-1}\\ {\hat{x}}_{k|k}^{\left(i+1\right)}={\hat{x}}_{k|k-1}+K_{k}^{\left(i+1\right)}\left(y_{k}-H_{k}{\hat{x}}_{k|k-1}\right)\\ P_{k|k}^{\left(i+1\right)}={\hat{P}}_{k|k-1}^{\left(i+1\right)}-K_{k}^{\left(i+1\right)}H_{k}{\hat{P}}_{k|k-1}^{\left(i+1\right)} \end{array}\right.$$

Equation (47) gives the result of the end of the iterative process.

(47) $$\left\{ \begin{array}{l} {\hat{x}}_{k|k}={\hat{x}}_{k|k}^{\left(N\right)},P_{k|k}=P_{k|k}^{\left(N\right)}\\ {\hat{m}}_{k|k}={\hat{m}}_{k|k}^{\left(N\right)},{\hat{M}}_{k|k}={\hat{M}}_{k|k}^{\left(N\right)}\\ {\hat{v}}_{k|k}={\hat{v}}_{k|k}^{\left(N\right)},{\hat{V}}_{k|k}={\hat{V}}_{k|k}^{\left(N\right)} \end{array}\right.$$

**Figure 5:**
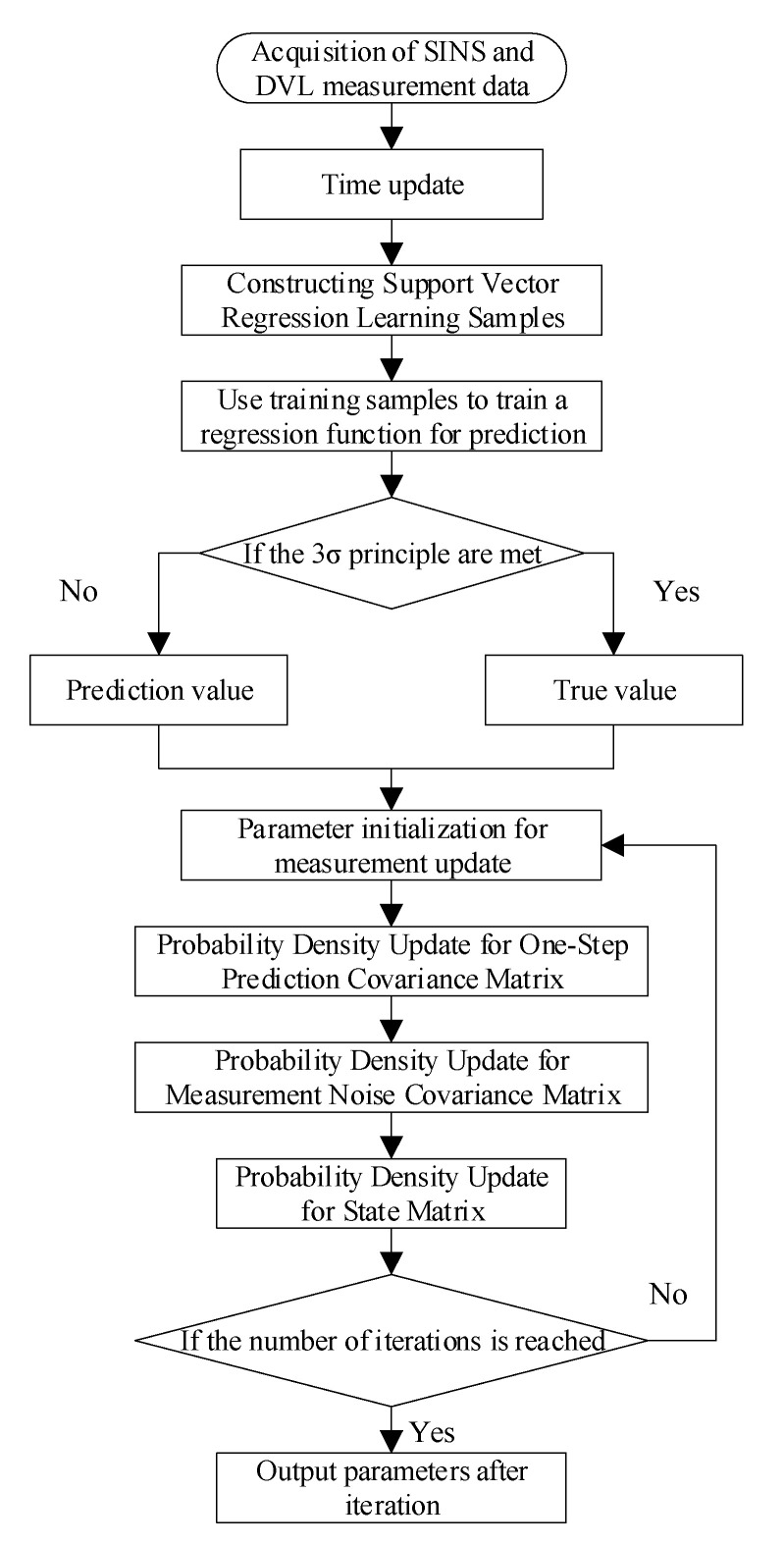
Process flow support vector regression assisted adaptive filter.

The specific flow chart of the adaptive filter assisted by support vector regression is given as Figure 5.

## 4. Experimental Results and Disscusions

### 4.1. Simulation Analysis

In order to verify the effectiveness of the method proposed in this paper, a simulation experiment is designed. Firstly, the trajectory of the AUV is simulated. Here, the complex changes of the underwater environment are simulated, and the outliers in the DVL measurement process are simulated. We assume that the actual probability distribution of the measurement noise is in the form of a mixed Gaussian noise distribution as follows

(48) $$\rho =\left(1-\alpha \right)N\left(0,\;R_{c}\right)+\alpha N\left(0,\;R_{p}\right)$$

In the above formula, α is the noise pollution ratio, usually taken as α = 0.1. $R_{c}$ is the measurement noise covariance matrix of the DVL output velocity information, and $R_{p}$ is the measurement noise covariance matrix of the DVL output polluted. Generally, there is $R_{p}=\lambda R_{c}$, where λ is the magnification, usually λ=200. The total simulation time is 1800 s. To evaluate the robustness of the filter, DVL velocity measurement outliers are added every 600 s. The gyroscope constant biases along three axes of body frame are $0.01^{{}^{\circ}}/h$, and angular random walks are $0.01^{{}^{\circ}}/\sqrt{h}$. The constant drift of the accelerometer are 100 μg, and the velocity random walks are $100\;\mu g/\sqrt{Hz}$. The initial misalignment errors in the three directions are all set as $3^{\prime }$. The output frequencies of the Inertial Measurement Unit (IMU) and DVL are 200 Hz and 1 Hz. In the simulation, it is assumed that the initial position of the underwater vehicle is latitude $34.24^{\circ }\;N$, longitude is $108.90^{\circ }\;E$, and height is set to 100 m. The initial value of measurement noise is set to $R=diag\left(\left[0.1^{2}\;0.1^{2}\;0.1^{2}\right]\right)$.The initial value of the state covariance matrix is set to

$$P=diag\left({\left[\left[0.1^{\circ }\;0.1^{\circ }\;0.1^{\circ }\right]\;\left[0.1\;0.1\;0.1\right]\;\left[0.1^{\circ }/h\;\;0.1^{\circ }/h\;\;0.1^{\circ }/h\right]\;\left[0.1^{\circ }/h\;0.1^{\circ }/h\;0.1^{\circ }/h\right]\;\left[10^{-4}\;10^{-4}\;10^{-4}\right]*g\right]}^{2}\;\right)$$

To compare the performance of different filters We selected six filters including our method for comparison. They are the traditional Kalman filter (KF), the strong tracking filter (STF) first proposed in [44], the robust Kalman adaptive filter (HRAKF) proposed in [34], A new type of Variational Bayesian Adaptive Kalman Filter (VBAKF) [36], Robust Kalman Filter (OD-RSTKF) based on Outlier Detection and Student’s T Distribution Modeling proposed in [35], and the proposed method (SVR-VBAKF). The relevant parameter settings of the above-mentioned filters for comparison are consistent with those in the literature. In the method proposed in this paper, the length of the sliding window is set to N=20, the model order is m=6. The parameters in the Variational Bayes filter are selected as τ=5, the forgetting factor is ρ=0.99, and the number of fixed-point iterations is 3. Indicators for evaluating filter performance mainly choose the root mean square error of heading angle error, velocity error and position error as the evaluation criteria. A total of 50 Monte Carlo simulation experiments are performed.

**Figure 6:**
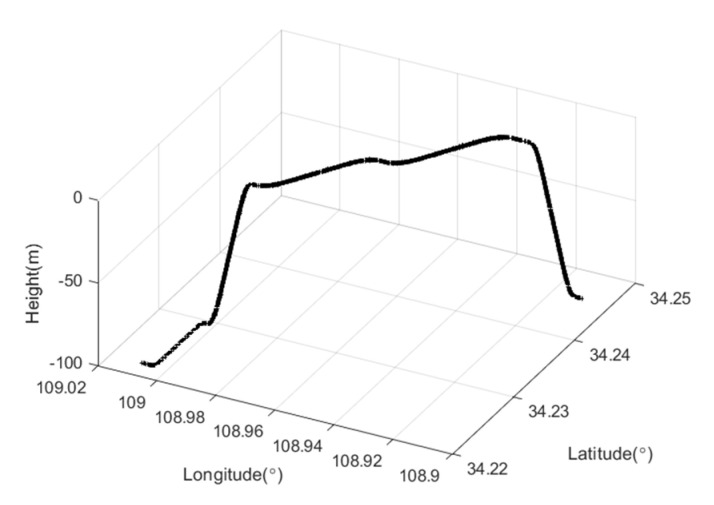
Curve of moving trajectory.

The schematic diagram of the trajectory of the simulation experiment is given as Figure 6.

**Figure 7:**
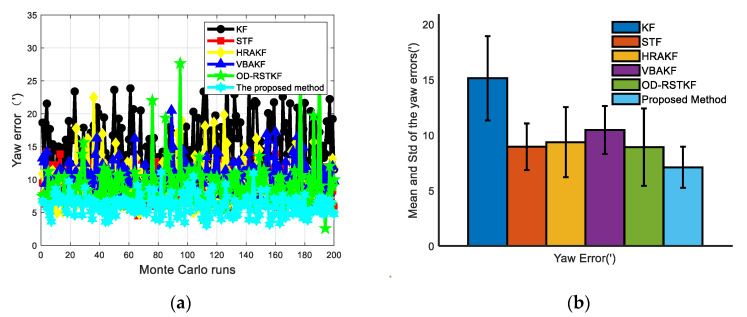
Heading error results of various filters (**a**) Heading angle error of various filters under 50 Monte Carlo simulations; (**b**) Mean and STD of heading angle errors of various filters under 50 Monte Carlo simulations.

**Figure 8:**
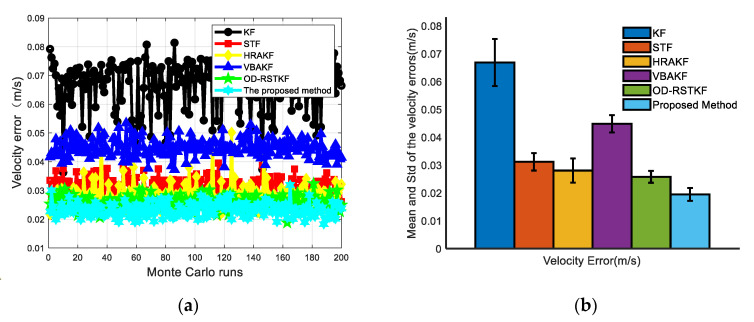
Velocity error results of various filters (**a**) Velocity angle error of various filters under 50 Monte Carlo simulations; (**b**) Mean and STD of velocity errors of various filters under 50 Monte Carlo simulations.

**Figure 9:**
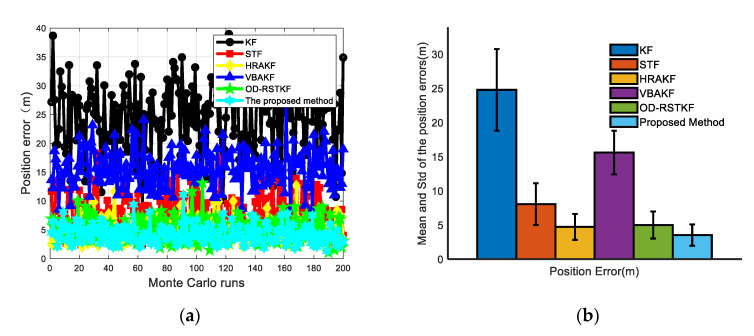
Position error results of various filters (**a**) Position angle error of various filters under 50 Monte Carlo simulations; (**b**) Mean and STD of position errors of various filters under 50 Monte Carlo simulations.

In the simulation process, the trajectory of the underwater vehicle has experienced a total of basic motion processes such as diving, floating, turning left and right, acceleration and deceleration. The heading angle error, velocity error and position error curves of 50 Monte Carlo simulation experiments are given as Figure 7, Figure 8 and Figure 9.

The mean values of heading, velocity and position mean square errors for the above six filters are given in Table 1 and Table 2. It is not difficult to see that compared with the traditional methods such as KF, the mean square error of the position has increased by 86.64%, 57.70%, 27.07%, 77.21% and 28.82%. The mean square error of heading has increased by 53.08%, 20.69%, 24.24%, 32.13%, and 20.32%. The average velocity error increased by 70.95%, 37.62%, 30.71%, 56.69%, and 24.51%. At the same time, in order to compare the stability of the filter, we use the standard deviation of the mean square error for evaluation. In the case of 200 Monte Carlo simulations, the robust adaptive method proposed in this paper has the minimum standard deviation and mean value in heading error, velocity error and position error, which fully shows that the method proposed in this paper has better filtering effect and stability. It can be seen from Table 3 that the robust adaptive filtering method will take a longer time, but the method proposed in this paper brings higher navigation accuracy. From the experimental results, it is easy to find that the traditional Kalman filter method is significantly less effective in dealing with thick-tailed noise and outliers, which shows that it is very important to improve the adaptability and robustness of the filter. The VBAKF method is a new type of adaptive filtering method, and does not pay too much attention to the robustness, but from the experimental results, the method still has a certain robustness, but its effect is obviously not as good as other methods. Several adaptive robust filtering methods selected for comparison have certain effects, especially the adaptive filtering algorithm assisted by support vector machine proposed in this paper has more obvious advantages.

### 4.2. Shipboard Test Analysis

**Figure 10:**
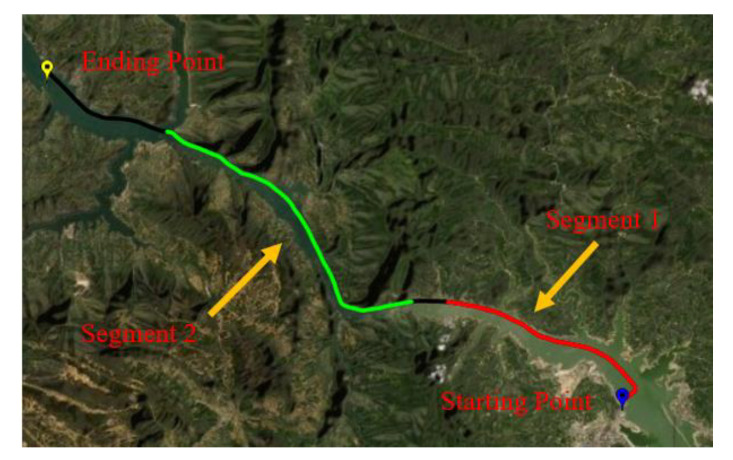
The trajectory of experiment.

In order to further verify the effect of the method proposed in this paper, the corresponding ship-borne test was carried out. The test ship was equipped with a set of equipment for IMU and DVL. The experimental time was 9000 s. The parameters of the corresponding equipment used in the test are shown in the Table 4 and Table 5:

For comparison, we divided the entire data into two segments. Each segment of data lasts for 3600 s, and the performance of several robust adaptive filtering methods proposed are compared through two data segments to verify the effectiveness of the method proposed in this paper. The corresponding trajectories of the two data segments are given as Figure 10.

**Figure 11:**
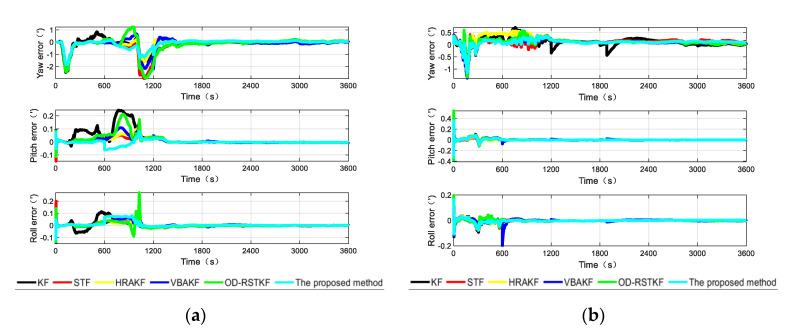
The attitude angle errors from different algorithms in two segments (**a**) The heading angle error of different algorithms in the first test; (**b**) The heading angle error of different algorithms in the second test.

**Figure 12:**
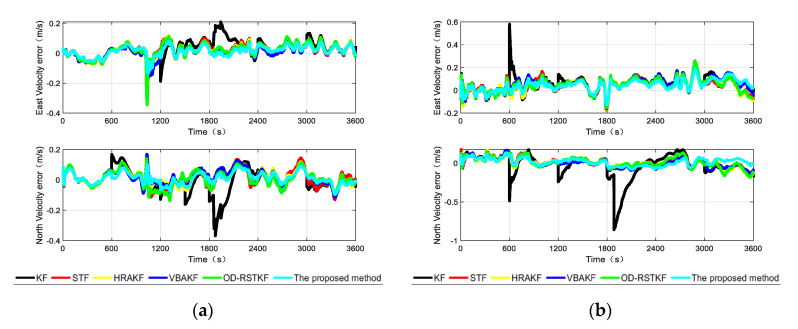
The velocity errors from different algorithms in two segments (**a**) The velocity error of different algorithms in the first test; (**b**) The velocity error of different algorithms in the second test.

**Figure 13:**
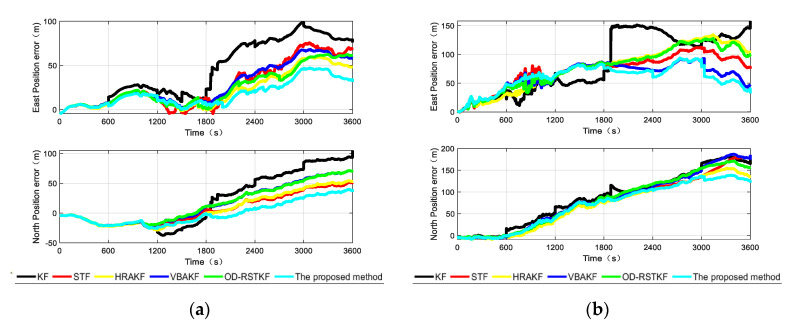
The position errors from different algorithms in two segments (**a**) The position error of different algorithms in the first test; (**b**) The position error of different algorithms in the second test.

After the experimental trajectories are given, the performance of the filtering algorithms will be compared. The initial parameter settings of the filter are the same as those of the simulation conditions, and the combined navigation attitude, speed and position error curves of the two data segments are given as Figure 11, Figure 12 and Figure 13.

According to the analysis of the error curves, in the verification of the first set of data, the oscillation amplitude and maximum error of the method proposed in this paper are the smallest within the first 600 s. About 600 s, the pitch angle error and roll angle error of the proposed method are reduced compared with other robust adaptive methods. Because the initial carrier is static and the speed is particularly low, the effect of using SVR method to detect small outliers has decreased, and it has not even been completely detected. Other robust adaptive methods will process outliers through innovation in each measurement update stage and adjust the measurement noise covariance matrix, so the effect will be better than that of this method. From the position error curves, the effect of traditional Kalman filter is obviously poor because there will be wild values in each section of DVL data. It cannot be denied that other robust adaptive Kalman filters have good effects on the suppression of outliers. Moreover, the method in this paper is not optimal at every moment. There may be the influence of the randomness of the filter, but the two segments of data are about 1800 s. Because the error caused by the wild value is very large, after being processed by different methods, the step change of the position error is well suppressed, and the method proposed in this paper has better positioning accuracy. It shows that the method in this paper can deal with outliers more thoroughly and has stronger robustness.

In the Table 6, it is not difficult to find that the method proposed in this paper has a better combined navigation effect than other methods, and this effect is more obvious in the position error. In the segment 1, compared with the traditional KF method, STF method, HRAKF method, VBAKF method and OD-RSTKF method, the position mean square error of this method is reduced by 59.83%, 33.98%, 25.68%, 40.49% and 36.51%. The mean square error of speed is reduced by 40.84%, 19.03%, 15.36%, 14.54% and 8.32%. In the segment 2, compared with the traditional KF method, STF method, HRAKF method, VBAKF method and OD-RSTKF method, the position mean square error of this method is reduced by 26.60%, 9.53%, 15.23%, 12.16% and 19.24%. The mean square error of speed is reduced by 54.98%, 15.31%, 15.21%, 19.23% and 15.91%. From the heading error results, it is obvious that the method proposed in this paper has a faster convergence of the attitude curve under the influence of outliers and non-Gaussian noise. It can be seen from the above two segments that the error of the second segment is greater than that of the first, because the second segment of data is purely dynamic data, while the first segment has static data. However, the SVR-VBAKF proposed in this paper has obvious suppression of position error and velocity error, and the performance of heading error is not much different from other methods, but it is obviously better than the traditional Kalman filter method. In terms of running time, this new method takes longer because of the use of machine learning to make predictions. At the same time, the average calculation time of several methods is not much different, but in exchange for higher navigation accuracy.

It can be seen from the above navigation error results that in some time periods, the traditional Kalman filter shows a step trend, this is because the DVL is prone to outliers in the measurement process, which leads to poor robustness of the integrated navigation system, and at the same time, due to the influence of process noise and measurement noise, the effect of the integrated navigation is also deteriorated. Compared with the filter based on Variational Bayes principle and strong tracking filter, the method proposed in this paper has better robustness and adaptability. Compared with HRAKF and OD-RSTKF method, Process external sensor information directly in use, taking advantage of the small sample data training of the SVR algorithm, and adapting the threshold value through the statistical principle, the effective prediction of sensor information is realized. At the same time, no other methods are introduced in the filtering framework, which avoids complex operations inside the filter, improves the stability of the filter, and hardly produces errors caused by inaccurate matrix decomposition. Especially in the case of obvious outlier processing, it has stronger advantages.

## 5. Conclusions

In this paper, in order to effectively improve the adaptability and robustness of the SINS/DVL integrated navigation system filter, an adaptive filtering method based on support vector regression assistance (SVR-VBAKF) is studied. This method considers the robustness and adaptive processing methods of the integrated navigation system separately. Firstly, by processing the external measurement information, the original filtering framework is changed, which effectively improves the robustness and stability of the filter. At the same time, an adaptive filter based on Variational Bayesian method that can deal with imprecise process and measurement noise is used to deal with thick-tailed noise to improve the adaptiveness of the filter. This method also has certain robustness, but the processing effect on outliers is poor. Therefore, the role of the adaptive filter can be better played after removing outliers by external means. Compared with several existing algorithms, the method proposed in this paper has stronger robustness and adaptability when the motion carrier has obvious outlier changes, and has higher navigation accuracy, which is proved by simulation and on-board experimental tests. Further work will focus on the practical application of underwater vehicles and the short-term failure of the DVL signal.

## Figures and Tables

**Table 1:** Mean for Yaw, Velocity and Position RMSE.

Algorithm	Yaw Error (′)	Velocity Error (m/s)	Position Error (m)
KF	15.1338	0.0668	24.7637
STF	8.9520	0.0311	8.4056
HRAKF	9.3707	0.0280	4.8771
VBAKF	10.4607	0.0448	15.6017
OD-RSTKF	8.9104	0.0257	4.9974
SVR-VBAKF	7.0995	0.0194	3.5557

**Table 2:** STD for Yaw, Velocity and Position RMSE.

Algorithm	Yaw Error (′)	Velocity Error (m/s)	Position Error (m)
KF	3.8072	0.0084	5.9700
STF	2.5062	0.0031	3.0762
HRAKF	3.1153	0.0043	1.6891
VBAKF	2.1701	0.0031	3.1848
OD-RSTKF	3.4967	0.0025	1.9814
SVR-VBAKF	1.8566	0.0021	1.5635

**Table 3:** Monte Carlo Simulation Single Computing Time.

Algorithm	Single Computing Time (s)
KF	$2.577\times 10^{-2}$
STF	$2.508\times 10^{-2}$
HRAKF	$3.977\times 10^{-2}$
VBAKF	$2.821\times 10^{-2}$
OD-RSTKF	$6.173\times 10^{-2}$
SVR-VBAKF	$5.562\times 10^{-2}$

**Table 4:** The performance of IMU.

Performance	Gyroscope	Accelerometer
Measuring range	±200 deg/s	±15 g
Update Rate	200 Hz	200 Hz
Constant Drift	<0.02 deg/h(1σ)	<50 μg(1σ)

**Table 5:** The performance of DVL.

Performance	DVL
Accuracy Level	0.5% V±0.5 cm/s
Speed range	−5.14~10.28 m/s
Update Rate	1 Hz
Frequency	300 kHz
Bottom tracking depth	300 m

**Table 6:** Mean square errors of yaw, velocity, position and single step running time of each group of data under different algorithms.

Algorithm	Segment	Yaw Error (′)	Velocity Error (m/s)	Position Error (m)	Computing Time (s)
KF	Segment 1	0.5232	0.1043	74.5670	0.0467
	Segment 2	0.2386	0.1819	143.6689	
STF	Segment 1	0.6629	0.0762	45.3782	0.0478
	Segment 2	0.1783	0.0967	116.5778	
HRAKF	Segment 1	0.4132	0.0729	40.3082	0.0503
	Segment 2	0.2549	0.0966	124.4007	
VBAKF	Segment 1	0.4514	0.0722	50.3451	0.0480
	Segment 2	0.1752	0.1014	120.0570	
OD-RSTKF	Segment 1	0.6997	0.0673	47.1850	0.0520
	Segment 2	0.1918	0.0974	130.5579	
SVR-VBAKF	Segment 1	0.4166	0.0617	29.9567	0.0528
	Segment 2	0.1812	0.0819	105.1596	

## Data Availability

Data sharing not applicable. No new data were created or analyzed in this study. Data sharing is not applicable to this article.
